# Supplementary figures and images for: A new point mutation in the HC-Pro of potato virus Y is involved in tobacco vein necrosis
Source: PLoS One. 2024 May 9;19(5):e0302692. doi: 10.1371/journal.pone.0302692 (PMC11081373; doi:10.1371/journal.pone.0302692)

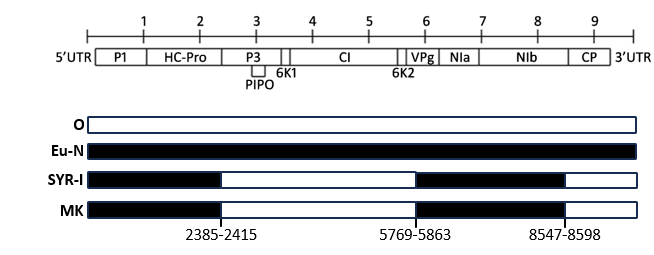

Supplement: S1 Fig — The organization of the PVY genome (ca. 9.7-kb long) is reported on the top, where individual cistrons are presented as rectangles with corresponding protein names. The putative parental sequences of PVY-MK, i.e. PVY group O in white and PVY group Eu-N in black, are shown below the PVY genome. The recombinant structure of group SYR-I [13] is also reported to show its similarity with that of PVY-MK. (TIF) [file pone.0302692.s001.tif]
