# Supplementary material for: A new point mutation in the HC-Pro of potato virus Y is involved in tobacco vein necrosis
Source: PLoS One. 2024 May 9;19(5):e0302692. doi: 10.1371/journal.pone.0302692 (PMC11081373; doi:10.1371/journal.pone.0302692)
Supplement: S1 Table — (DOCX) [file pone.0302692.s002.docx]

**Table S1.** Primers used for genome amplification and sequencing of PVY-MK

| **No.** | **Oligo name** | **Sequence 5’-> 3’** | **Tm [°C]** | **Expected amplicon** | **Reference** |
| --- | --- | --- | --- | --- | --- |
| 1 | PVY1F | AATTAAAACAACTCAATACAAC | 50 | 1359 | This study |
|  | PVY2R | CCGGTTCAGTTAAGTGCTCT |  |  | This study |
| 2 | PVY3F | GATCGCTTTGTGCATGTCAA | 52 | 1384 | This study |
|  | PVY4R | GCGATGCTATCATAGCTATGG |  |  | This study |
| 3 | PVY5F | GGTAAGGTTGTGGATTAATGA | 54 | 1198 | This study |
|  | PVY6R | TTTCCAGACCCAACAGCTCCC |  |  | This study |
| 4 | PVY7F | GGGAGCTGTTGGGTCTGGAAA | 56 | 1331 | This study |
|  | PVY8R | CTGATGGACGATGCTCGAATGC |  |  | This study |
| 5 | PVY9F | GCTCCACGAAATGCTTTGGG | 52 | 1359 | This study |
|  | PVY10R | TCTGCCTTTGATCGGTAAAAC |  |  | This study |
| 6 | PVY11F | CATTCAGAGTGAAGAATTTGC | 52 | 1351 | This study |
|  | PVY12R | GCTTCTGATGATGAGAACAGC |  |  | This study |
| 7 | PVY13F | CACAATTCGATAGTTCACTCACC | 54 | 942 | This study |
|  | PVY14R | GGCACAGTATGAGTTCCAGATG |  |  | This study |
| 8 | S | GGNAAYAAYAGYGGNCARCC | 47 | 1766* | Chen et al., 2001 |
|  | M4 | GTTTTCCCAGTCACGAC |  |  | Chen et al., 2001 |
|  | M4-T | GTTTTCCCAGTCACGACTTTTTTTTTTTTTTT |  |  | Chen et al., 2001 |
|  |  |  |  |  |  |

*without poly-A tail

**References for table S1**

Chen, J.; Chen, J.; Adams, M.J. A universal PCR primer to detect members of the Potyviridae and its use to examine the taxonomic status of several members of the family. *Arch. Virol*., **2001**, 146, 757-766. doi: 10.1007/s007050170144. PMID: 11402861.
